# Active Smoking and Risk of Metabolic Syndrome: A Meta-Analysis of Prospective Studies

**DOI:** 10.1371/journal.pone.0047791

**Published:** 2012-10-17

**Authors:** Kan Sun, Jianmin Liu, Guang Ning

**Affiliations:** 1 Key Laboratory for Endocrine and Metabolic Diseases of Ministry of Health, Rui-Jin Hospital, Shanghai Jiao Tong University School of Medicine, E-Institute of Shanghai Universities, Shanghai, China; 2 Shanghai Clinical Center for Endocrine and Metabolic Diseases, Shanghai Institute of Endocrine and Metabolic Diseases, Department of Endocrinology and Metabolism, Rui-Jin Hospital, Shanghai Jiao Tong University School of Medicine, Shanghai, China; Fundación para la Prevención y el Control de las Enfermedades Crónicas No Transmisibles en América Latina (FunPRECAL), Argentina

## Abstract

**Background:**

Epidemiological evidence suggests that smoking has been associated with emergence of metabolic syndrome. However, data on this issue are inconsistent and controversial. We therefore conducted a meta-analysis to examine the association between smoking and metabolic syndrome.

**Methodology and Principal Findings:**

We searched the Medline, Embase and the Cochrane Library database up to March 2012 to identify prospective cohort studies related to smoking and metabolic syndrome. Reference lists of retrieved articles were also reviewed. Summary effect estimates were derived using a random-effects model and stratified by gender, smoking dose, follow-up duration and geographical area. Primary analysis of 13 studies involving 56,691 participants and 8,688 cases detected a significant positive association between active smoking and risk of metabolic syndrome (pooled relative risk [RR] 1.26, 95% CI: 1.10–1.44). Estimates of effects were substantially consistent in the stratified analyses. In the dose-response analysis, risk of metabolic syndrome was stronger for active male smokers (pooled RR 1.34, 95% CI: 1.20–1.50) than it was for former male smokers (pooled RR 1.19, 95% CI: 1.00–1.42), and greater for heavy smokers (pooled RR 1.42, 95% CI: 1.27–1.59) compared with light smokers (pooled RR 1.10, 95% CI: 0.90–1.35). No evidence of statistical publication bias was found (Egger' s test *P* = 0.227, Begg' s test *P* = 0.113).

**Conclusions:**

Active smoking is associated with development of metabolic syndrome. Smoking cessation appears to reduce the risk of metabolic syndrome.

## Introduction

The adverse effect of tobacco use on health has been well established for over half a century. Consumption of tobacco is one of the leading causes of avoidable death globally. It is estimated that more than 8 million people may die from smoking and its related causes by 2030 [1]. Metabolic syndrome includes the constellation of various metabolic abnormalities and confers an increased risk for diabetes and cardiovascular diseases. It is estimated that the prevalence of metabolic syndrome in adult was around 20-25% all over the world [2].

The association of tobacco use with the onset of metabolic syndrome has been recognized in the past decade. Cigarette smoking has been proven to play a role in emergence of various components of metabolic syndrome and hence could lead to occurrence and progression of the disease through multiple mechanisms. However, available data from epidemiological studies on this issue are inconsistent and controversial. The positive correlation between smoking and metabolic syndrome is significant in some but not all studies [3], [4]. One study conducted among Turkish women even found a protective effect of smoking on metabolic syndrome [5]. Different definitions of metabolic syndrome and individual baseline information of the study population might lead to inconsistent results on this issue.

Previously existing literature and primary analyses on the association of smoking with the onset of metabolic syndrome did not complete a meta-analysis of data sources, and statistical power was inadequate and insufficient in these recent studies. As a result, the aim of the present meta-analysis is to assess the relationship between smoking and metabolic syndrome and to obtain a quantitative estimate of the risk.

## Methods

### Search strategy and study selection

We conducted a meta-analysis of the published works without language restrictions and in accordance with the Meta-analysis of Observational Studies in Epidemiology (MOOSE) guidelines [6]. We searched Medline, Embase, and the Cochrane Library from their inception to March 2012 and identified prospective studies that evaluated the effect of smoking on the risk of metabolic syndrome. The main search terms were “smoking” or “smoking cessation” or “tobacco” or “nicotine” or “cigarette” in combination with “metabolic syndrome” or “insulin resistance syndrome” or “syndrome X” with no restrictions. Reference lists from published original articles, and previous reviews were scanned for more relevant studies not identified in the databases search. Because of the high potential for intractable confounding and reverse causation, cross-sectional studies were excluded in this meta-analysis.

Studies were included in the meta-analysis if they met the following criteria: the study have a prospective cohort design; published quantitative estimates of the association between smoking and risk of metabolic syndrome in male or female; the endpoint of interest was incidence of metabolic syndrome; description of adjustment for potential confounders and had been adjusted at least for age [7]. Studies were excluded if the study selected a comparison group that was not nonsmokers; case-control design and cross-sectional design was used; study that examined other associations.

### Data extraction and quality assessment

Two of our reviewers independently evaluated all relevant articles and identified eligible studies from the databases. During data abstraction, differences and disagreements were resolved through discussion to come to an agreement. We used standardized data extraction forms to record the following information: last name of the first author, publication year, geographic region of original study, mean length of follow-up, number of cases and participants, measurement of exposure and outcome, unadjusted and adjusted risk of developing metabolic syndrome for active smokers compared with nonsmokers with corresponding 95% confidence interval (CI) and adjustment factors of interest. Data for the association between former smoking and risk of metabolic syndrome was extracted when possible. In case of multiple publications from one study population, data relating to the most complete publication was extracted and included in the analysis. Researchers with professional knowledge in this area were queried for the presence of unpublished reports. We did not contact authors of the primary studies for additional information. As the data analyses were rely mainly on the published results, methodological quality was very important of the included studies. Hence, a 9-scores system on the basis of the Newcastle-Ottawa Scale (NOS) was used to assess the quality of the included studies [8]. In this scoring system, each study included in the meta-analysis was judged on three broad perspectives: the selection of the study cases, the comparability of the study populations and the ascertainment of either the exposure or outcome of interest.

### Statistical analysis

Our primary analyses were focused on a comparison of the summary relative risk (RR) of metabolic syndrome in current smokers versus non-smokers. When studies presented results from various covariates analyses, we used the one adjusted for the main potential confounders, such as age, sex, body mass index (BMI), physical activity, alcohol consumption and weight change. The pooled estimates were calculated by averaging the natural logarithmic RR or hazard ratios or odds ratios (OR) weighted by their inverse of variance based on a fixed or random effects model within or between study variations, depending on the overall heterogeneity. Heterogeneity of effect size across studies was assessed by using Cochran's Q and the *I^2^* statistic [9], [10] and *P* value<0.10 or *I^2^* value>50% was considered to be heterogeneous. When substantial heterogeneity was detected, we calculated summary RR and their 95% CI with the method of DerSimonian and Laird in a random effects model [11]. If not, the pooled estimate was presented based on the fixed effects model by using the inverse variance method [12].

Nine studies defined a group of former smokers and therefore with a reference group defined as never smokers [5], [13], [14], [15], [16], [17], [18], [19], [20], but 4 studies classified the exposure variable by smokers and nonsmokers [21], [22], [23], [24], without information whether the nonsmoking group included former smokers. For this reason, we conducted a sensitivity analysis by including studies that used reference group as absolutely never smokers. Two long time duration follow-up studies (baseline survey in the 1970s) [17], [18] could not screen metabolic syndrome (one excluded diabetes and the other excluded diabetes and coronary heart disease at the baseline survey) for lacking measurements of metabolic syndrome components. Hence, we conducted a sensitivity analysis by removing the 2 studies and recalculated the combined estimate on remaining studies. To assess the influence of individual studies on the pooled result, we conducted a sensitivity analysis by omitting one study in each turn.

Subgroup analyses according to gender (male/female), amount of smoking (heavy, ≥20 cigarettes per day/light, <20 cigarettes per day), definition of metabolic syndrome (strict National Cholesterol Education Program [NCEP] III/other modified criteria compared with strict NCEP III definition), mean follow-up time (≥5 years/<5 years), geographical area (Asia/Europe/North America), and study quality (high, NOS scores ≥7/low, NOS scores<7) were used to assess the impacts of study characteristics on outcomes. Meta-regression analysis was used to investigate the influence of these variables on study heterogeneity across strata. We used the Begg' s adjusted rank correlation test and the Egger' s regression asymmetry test to detect publication bias and *P*>0.05 for both tests was considered to be no significant publication bias. All statistical analyses were performed using STATA version 11.0 (Stata Corp, College Station, TX, USA).

## Results

### Literature search

**Figure 1:**
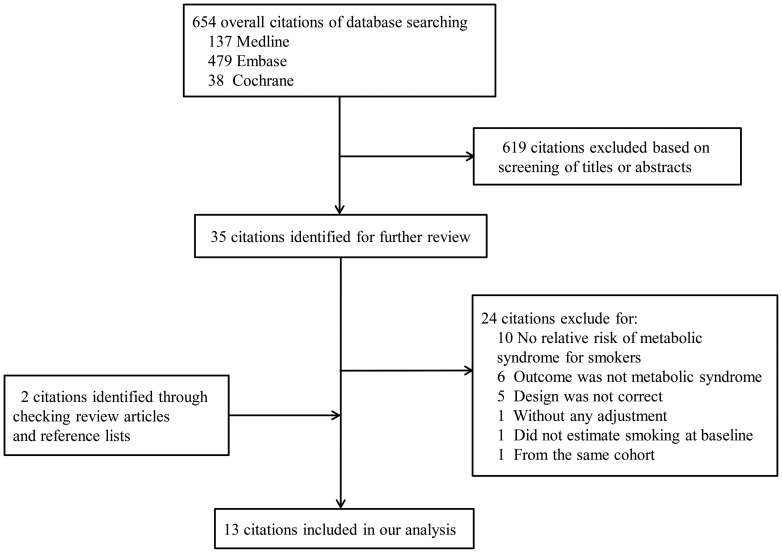
Flow diagram of study selection process.

The details of the literature search were presented in a flow diagram (Figure 1). We identified 654 citations (137 from Medline, 479 from Embase and 38 from Cochrane Library) with our electronic literature search. We excluded 619 citations after screening based on abstracts or titles. After this, 35 remained citations and 2 retrieved citations were full-text reviewed. Finally, 13 citations met the inclusion criteria and were included in the meta-analysis.

### Study characteristics and quality assessment

The characteristics and information of the included studies were shown in Table 1. The 13 selected studies contained 56,691 participants (ranging from 466 to 17,014) with 8,688 cases of metabolic syndrome from different populations (8 studies in Asia, 4 studies in Europe and 1 study in North America) and with varying length of the follow-up period (ranging from 1 to 28 years). Among the 13 included studies, 9 studies showed a significant positive correlation [13], [14], [15], [16], [17], [18], [19], [23], [24] and 1 study showed a significant inverse correlation between smoking and risk of metabolic syndrome [5]. No significant positive association was found in the remaining 3 studies [20], [21], [22]. Assessment of study specific quality scores from NOS system were summarized in Table 2. The median score of included studies was 7, with a range from 5 to 9, and 76.9% of the studies were identified as relatively high-quality.

**Table 1:** Characteristics of included studies of smoking and metabolic syndrome risk included in the meta-analysis.

						Metabolic syndrome incidence by smoking status (No./Total)			
Source	Participants	Age range (years)	Mean duration (years)	Definition of metabolic syndrome ^1^	New cases (incident, %)	Current	Former	Never	Adjusted Variables ^2^
Kim 2009 Korea [13]	Male: 4542	42	2.9	WHO Guidelines	482 (10.6)	179/1292	85/496	202/2528	age, weight, lifestyle status, the number of metabolic syndrome components and weight change
Yang 2008 Taiwan [21]	All: 9785 Male:4707 Female:5078	19–84	4	Modified NCEP III	1245 (12.7)	NA	NA	NA	age, education, alcohol consumption, occupational physical exertion and BMI
Nakanishi 2005 Japan [14]	Male: 2994	35–59 ^3^	7	Modified NCEP III	696 (21.9)	353/1494	143/585	160/915	age, family history of diabetes, alcohol consumption, and physical activity
Zhu 2011 China [15]	Male: 693	57	3	Modified NCEP III ^4^ and JCDCG	150 (21.7)	86/375	23/95	41/222	age, education level, HOMA-IR, insulin, alcohol intake, BMI and weight change
Kim 2007 Korea [16]	Male: 1578	20–59	2	ACE/AACE	218 (13.8)	NA/555	NA/436	NA/405	age, waist hip rate, aminotransferase, low-density lipoprotein cholesterol
Holme 2007 Norway [17]	Male: 6382	40–49 ^3^	28	Modified NCEP III	1597 (25.0)	767/2801	476/1979	354/1602	age, years of education, glucose, triglycerides, BMI, treated hypertension and systolic blood pressure
Wilsgaard 2007 Norway [22]	All: 17014 Male: 8546 Female: 8468	20–61	13.8	NCEP III	1942 (11.4)	NA	NA	NA	age, time of baseline examination, alcohol intake, coffee consumption, years of education and physical activity
Wannamethee 2006 UK [18]	Male: 3051	40–59	20	NCEP III	790 (25.6)	119/405	454/1686	228/952	age, BMI, dietary fat, carbohydrate, physical activity, alcohol consumption and social class
Onat 2006 Turkey [5]	All: 1961 Male: 947 Female: 1014	≥28	5.9	Modified NCEP III	472 (24.1)	NA	NA	NA	age, sex, physical activity grade and family income
Kawada 2009 Japan [19]	All: 2136	45.4	1	Modified NCEP III ^5^	135 (6.3)	NA/1006	NA	NA/1070	age, work history and HOMA-IR
Li 2010 Japan [23]	Male: 1897	35-60	3	AHA/NHLBI	285 (15)	125/660	64/488	94/740	age, BMI, physical activity, healthy eating behaviors and weight change
Puustinen 2011 Finland [24]	All: 466 Male: 185 Female: 281	NA	6.4	Modified NCEP III	101 (22)	NA/120	NA	NA	age, sex, socioeconomic status, use of alcohol, leisure time physical activity, high sensitivity C-reactive protein and psychological distress
Carnethon 2004 America [20]	All: 4192 Male: 1869 Female: 2323	24.9	13.6	NCEP III	575 (13.7)	NA/1203	NA/553	NA/2436	age, race, sex, education, BMI, physical activity, alcohol consumption, energy intake, crude fiber intake and weight change

1. WHO Guidelines, World Health Organization-West Pacific Region Guidelines; NCEP-ATP III, National Cholesterol Education Program's Adult Treatment Panel III; JCDCG, Chinese Joint Committee for Developing Chinese Guidelines on Prevention and Treatment of Dyslipidemia in Adults definition; ACE/AACE, American College of Endocrinology/American Association of Clinical Endocrinologists criteria; AHA/NHLBI, American Heart Association/National Heart, Lung and Blood Institute criteria.

2. BMI, body mass index; HOMA-IR, homeostasis model assessment of insulin resistance.

3. Age of all participants (include those with metabolic syndrome at the baseline); NA, not recorded or available.

4. We used the risk estimates from NCEP III definition in this study.

5. Kawada used Japanese criteria which is similar to NCEP criteria except for lower waist circumference.

**Table 2:** Assessment of Study Quality included in the meta-analysis. ^1^

	Selection				Comparability ^2^		Outcome			Total Scores
Source	1	2	3	4	5A	5B	6	7 ^3^	8 ^4^	
Kim, 2009, Korea [13]	☆	☆	-	☆	☆	☆	☆	-	☆	7
Yang, 2008, Taiwan [21]	☆	☆	-	☆	☆	☆	☆	-	☆	7
Nakanishi, 2005, Japan [14]	-	☆	☆	☆	☆	☆	☆	☆	☆	8
Zhu, 2011, China [15]	☆	☆	☆	☆	☆	☆	☆	-	-	7
Kim, 2007,Korea [16]	-	☆	-	☆	☆	☆	☆	-	☆	6
Holme, 2007, Norway [17]	☆	☆	☆	-	☆	☆	☆	☆	-	7
Wilsgaard, 2007, Norway [22]	☆	☆	-	☆	☆	☆	☆	☆	☆	8
Wannamethee, 2006, UK [18]	☆	☆	-	-	☆	☆	☆	☆	☆	7
Onat, 2006, Turkey [5]	☆	☆	-	☆	☆	☆	☆	☆	☆	8
Kawada, 2009, Japan [19]	-	☆	-	☆	☆	☆	☆	-	☆	6
Li, 2010, Japan [23]	-	☆	-	☆	☆	☆	☆	-	-	5
Puustinen, 2011, Finland [24]	☆	☆	☆	☆	☆	☆	☆	☆	☆	9
Carnethon, 2004, America [20]	☆	☆	-	☆	☆	☆	☆	☆	☆	8

1. Representativeness of the exposed cohort; 2.Selection of the non-exposed cohort; 3.Ascertainment of exposure; 4.Demonstration that outcome of interest was not present at start of study; 5.Comparability of cohorts on the basis of the design or analysis; 6.Assessment of outcome; 7.Was follow-up long enough for outcomes to occur; 8.Adequacy of follow up of cohorts.

2. Studies that controlled for age received one score, whereas studies that controlled for other important confounders received an additional score.

3. Study with follow-up time>5 years was assigned one score.

4. Study with follow-up rate>75% was assigned one score.

### Overall analyses

**Figure 2:**
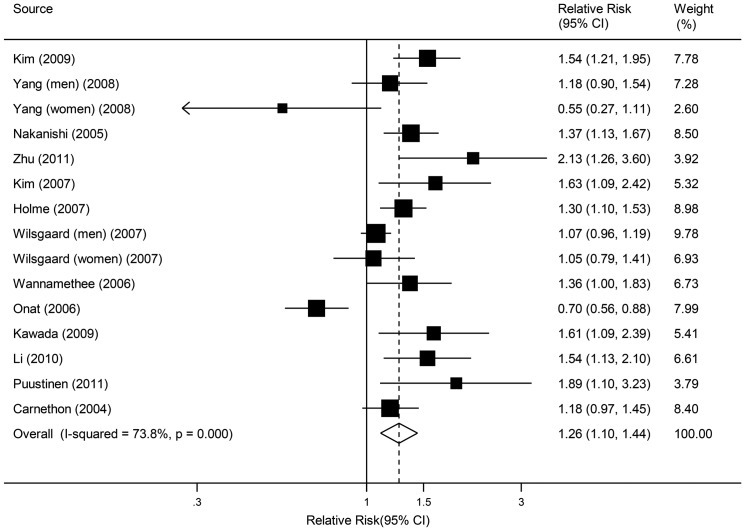
Relative risks of metabolic syndrome for active smokers compared with nonsmokers.

**Figure 3:**
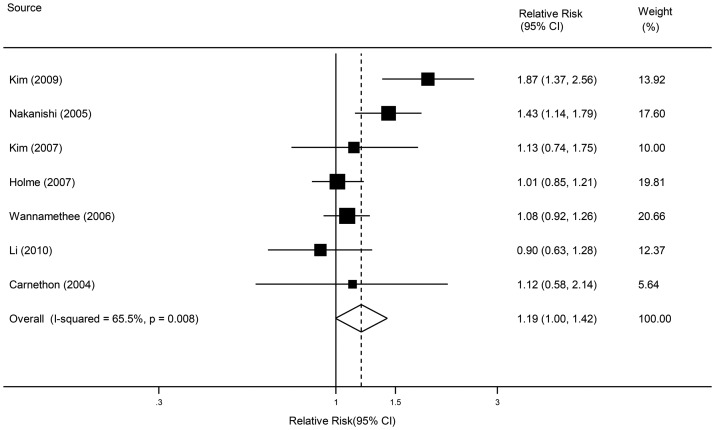
Relative risks of metabolic syndrome for former smokers compared with nonsmokers in male.

In overall analysis of the 13 selected studies, active smoking was associated with increased risk of metabolic syndrome (Figure 2, pooled RR 1.26, 95% CI: 1.10–1.44). Statistically significant evidence of heterogeneity was found across studies (*P*<0.001, *I^2^* = 73.8%). There was no indication of publication bias either from the result of Egger's test (*P* = 0.227) or from the Begg's test (*P* = 0.113). We performed the pooled estimates in former male smokers as there was only one study with reported risk of metabolic syndrome for former female smokers (RR 0.94, 95% CI: 0.66–1.36) [20]. Compared with nonsmokers, a borderline significant increased risk of metabolic syndrome was found in former male smokers (Figure 3, pooled RR 1.19, 95% CI: 1.00–1.42).

### Subgroup analyses

Subgroup analyses according to a number of characteristics to explore causes of the study heterogeneity were shown in Table 3. The result of increased metabolic syndrome risk in active smokers was consistently found in the stratified analyses except when stratified by gender. Male smokers (pooled RR 1.34, 95% CI: 1.20–1.50) seemed to have a substantially greater metabolic syndrome risk as compared with female smokers (pooled RR 0.85, 95% CI: 0.60–1.21).Moreover, the difference was statistically significant in meta-regression analysis (*P* = 0.02). In the dose-response analysis, compared with light smokers (pooled RR 1.10, 95% CI: 0.90–1.35), the risk of metabolic syndrome was stronger and more significant in heavy smokers (pooled RR 1.42, 95% CI: 1.27–1.59). As shown in Table 3, modified definitions of metabolic syndrome appear to have some influence on the consequences, however, definition by strict NCEP III (pooled RR 1.11, 95% CI: 1.02–1.21) and all other definitions (pooled RR 1.32, 95% CI: 1.09–1.60) reported a significant association between smoking and risk of metabolic syndrome. In the stratified analysis by mean follow-up time, the positive association between smoking and metabolic syndrome was slightly weaker if follow-up duration more than 5 years (pooled RR 1.16, 95% CI: 0.99–1.35). In a study carried out in North America, no significant evidence of association between smoking and risk of metabolic syndrome was found (RR 1.18, 95% CI: 0.97–1.44). However, such correlation was significant in studies performed in Asia (pooled RR 1.29, 95% CI: 1.01–1.64) and Europe (pooled RR 1.21, 95% CI: 1.04–1.41). When stratified by study quality according to NOS scoring system, both high quality studies (pooled RR 1.20, 95% CI: 1.04–1.38) and low quality studies (pooled RR 1.58, 95% CI: 1.29–1.95) showed consistent positive association between smoking and risk of metabolic syndrome.

**Table 3:** Stratified Analyses of Pooled Relative Risks of Metabolic Syndrome for Smoker.

Group	NO. of studies	RR (95% CI)	*P*_for heterogeneity_	*I* ^2^ (%)	*P*_for meta-regression_
Gender					0.02
Male	10	1.34 (1.20–1.50)	0.02	54.9	
Female	4	0.85 (0.60–1.21)	0.02	71.2	
Amount of smoking					0.07
Heavy (≥20 cigarettes/d)	3	1.42 (1.27–1.59)	0.02	68.9	
Light (<20 cigarettes/d)	3	1.10 (0.90–1.35)	0.31	16.5	
Definitions of metabolic syndrome					0.45
Strict NCEP III	3	1.11 (1.02–1.21)	0.44	0.00	
Other	10	1.32 (1.09–1.60)	0.00	78.1	
1 risk factor modified^*^	4	1.37 (1.22–1.55)	0.49	0.00	
2 risk factors modified	4	1.36 (1.00–1.84)	0.02	65.5	
>2 risk factors modified	2	1.04 (0.48–2.25)	0.00	95.5	
Mean follow-up time					0.18
≥5 years	7	1.16 (0.99–1.35)	0.00	76.8	
<5 years	6	1.44 (1.18–1.75)	0.05	52.1	
Geographical area					0.77
Asia	8	1.29 (1.01–1.64)	0.00	81.6	
Europe	4	1.21 (1.04–1.41)	0.07	53.9	
North America	1	1.18 (0.97–1.44)	-	-	
Study quality					0.17
High (NOS scores≥7)	10	1.20 (1.04–1.38)	0.00	75.8	
Low (NOS scores<7)	3	1.58 (1.29–1.95)	0.97	0.00	

* Modified criteria used to diagnose metabolic syndrome as compared with strict NCEP III definition.

### Sensitivity analyses

A sensitivity analysis was carried out by including 9 studies in which the comparison with a reference group defined as strictly never smokers (no former smokers) and the pooled RR was 1.32 (95% CI: 1.10–1.59). By removing the 2 studies which could not biologically screen metabolic syndrome at the baseline, the combined results showed that positive relation between smoking and risk of metabolic syndrome continued to be significant 1.25 (95% CI: 1.07–1.46). Similarly, we investigated the influence of a single study on the overall risk estimate by excluding one study at a time. The combined RR of overall risk estimates were consistent and without apparent fluctuation, with a range from 1.23 (95% CI: 1.08–1.40) to 1.31 (95% CI: 1.18–1.46). Moreover, studies used RR or OR to calculate the association showed a coincident outcome, with a pooled value of 1.13 (95% CI: 1.04–1.22) and 1.41 (95% CI: 1.13–1.77), respectively.

## Discussion

Findings of the present meta-analysis show a clear link between smoking and risk of metabolic syndrome. Based on data from 13 prospective cohort studies (56,691 participants; 8,688 incident cases of metabolic syndrome), active smokers have a 26% increased risk of metabolic syndrome compared with nonsmoking individuals. The combined estimate of our primary analysis was strong across multiple sensitivity analyses and without significant publication bias.

A previous study has shown the risk for the development of metabolic syndrome was significantly lower in female heavy smokers [5]. However, when reporting a beneficial effect of such a harmful exposure as smoking, we cannot be too cautious to draw a conclusion. In the current analysis, our results showed no sign of significance protective effect of smoking on metabolic syndrome in female. Even though, the present analyses might still have underestimated the true risk of metabolic syndrome in female smokers. At first, it will take time to observe the full effect of smoking on metabolic syndrome risk in female. On one hand, both the proportion of heavy smokers and the mean daily consumption of cigarettes are generally lower in female smokers [25]. On the other hand, as compared to male smokers, female smokers are usually younger and not so common, at least in some areas of the world [26]. Moreover, more female smokers than male conceal their smoking habit which will give rise to the misclassification of the smoking exposure and attenuation of metabolic syndrome risk of the present analyses [7], [27].

A clear interaction between susceptibility and dose admits of a simple explanation in establishing causation of any environmental factor [28]. In the present meta-analysis, we found a dose-response relationship between smoking and risk of metabolic syndrome, with stronger correlation for heavy smokers than light smokers. The gathering of other risk factors in heavy smokers and the incomprehensive data recording of included studies can cause such relationship. In addition, the most effective therapeutic way for smokers to attenuate the adverse effects of metabolic disorders and cardiovascular disease is to stop smoking. This is consistent with our finding that risk of metabolic syndrome in male was stronger for current smokers than it was for former smokers. Reduction of the triglyceride and improved insulin sensitivity and high density lipoprotein may adequate to account for the benefit of stop smoking [29]. Although somewhat arbitrary, we preliminarily inferred that smoking cessation may help to reduce the incidence of metabolic syndrome, at least for male smokers. Nevertheless, association between smoking and metabolic syndrome was less strong in studies with follow-up more than 5 years. This is probably because 2 [17], [18] of the 7 studies in this subgroup could not biologically exclude metabolic syndrome in their 1970s baseline survey. However, in order to obtain a long-range and more comprehensive assessment of the association between smoking and metabolic syndrome, our primary analysis was carried out by including those studies. Further sensitive analysis by removing the 2 studies showed that the association is still positive and significant.

It is biologically plausible that smoking could increase the risk of metabolic syndrome. Exposure to tobacco has been found to play a core role in emergence of many metabolic disorders. Certain evidences exist that smoking can increase blood pressure, waist circumference, triglycerides, and reduce high density lipoprotein cholesterol. Moreover, compared with nonsmokers, active smokers usually have more serious insulin resistance and hyperinsulinemia, which may increase the risk of type 2 diabetes [30]. The effect of cigarette smoking on glucose and lipid metabolism may partly attribute to stimulation of sympathetic nervous system [31] and increase of circulating insulin-antagonistic hormones levels, such as cortisol and growth hormone [32]. Elevated plasma cortisol concentration and aggravated insulin resistance can cause localization of visceral fat mass and increase of waist circumference. In addition to the hormone disturbance, several lines of evidence have shown that endothelial dysfunction and its related arterial compliance reduction were more serious in smokers, which may have significant influence on insulin resistance and compensatory hyperinsulinemia [33], and hence could contribute to development and deterioration of metabolic syndrome [34]. Although smoking is known to be associated with weight loss in several studies [35], [36], clear evidence exist that smokers (especially heavy smokers) have higher BMI [37] and greater risk of abdominal fat accumulation than nonsmokers [38].

There were several limitations to this meta-analysis. First, the distinct definition of metabolic syndrome might provide biased estimates on the risk of the disease attributed to smoking. The concept of metabolic syndrome has existed for at least ten decades and a variety of definitions have been prescribed by organizations including the NCEP expert panel, American College of Endocrinology (ACE), American Heart Association (AHA), and the World Health Organization (WHO). Among these definitions, the Third Report of the NCEP' s Adult Treatment Panel (NCEP III) in the year 2001 provided a relatively simple and feasible approach for detecting components of metabolic syndrome, which make the definition widely used in both clinical and epidemiological studies. Actually, in the effort to early recognize high-risk groups and emphasize ethnic differences, criteria were modified with the attempt to best estimates for the hazards of metabolic syndrome. For example, studies carried out in Asia usually revised waist circumference to obtain a threshold more appropriate for Asian populations [39]. In the current analysis, metabolic syndrome was defined by rigorous NCEP III and modified NCEP III criteria in most of the included studies. Pooled RR of the metabolic syndrome were statistical significant in studies using strict NCEP III and other definitions.

Another limitation was the incomprehensive coverage of information of the present analysis. After all, the results in this analysis were only obtained data from Asia, Europe and North America and the majority of the included studies did not state if there were any racial differences among participants. The distinctions in regions and races among studies might have an influence on pooled risk estimates of metabolic syndrome. Nevertheless, stratified analysis showed that participants from both Asia and Europe had significant risk estimates. Moreover, in the present analysis of 13 studies, 10 studies were specific for cigarette smoking, while estimation of smoking from other tobacco products (cigars or pipes) were not clear in the rest 3 studies. Actually, no studies have investigated the effect of different types of smoking on the risk of metabolic syndrome and we cannot estimate whether such types of smoking are equivalent in their effects.

Third, evidence suggests that risk of metabolic syndrome could be biased by methodological features of the included studies. As the data on smoking status was based on self-declaration, risk of metabolic syndrome may be underestimated if participants did not reasonably reported the habits of cigarette smoking when completing questionnaires. However, studies have shown that the reliability of self-reported smoking habits is robust and highly correlated with exhaled carbon monoxide values [40]. In addition, the discordance between studies in definition of non-smokers may lead to selection bias. Some studies clearly defined non-smokers as people who had never smoked whereas others just dichotomized the exposure variable as smokers and non-smokers. We speculated that former smokers and never smoker were combined into one category as non-smoker in these studies. This form of classification will conceal the hazards of smoking and limit information about risk analyses for the two groups separately. Nevertheless, sensitivity analysis suggested that the results were stable in different definition of non-smokers.

Finally, substantial heterogeneity, partly due to diversity in gender and dose of exposure, was noted in current analysis. Such heterogeneity was not surprising because of unavoidable variations in study population and distinct adjustments across studies. Strong clustering of unhealthy lifestyles, such as little exercise, excessive drinking and poor diet, were more common in people smoking than in the general population. Not all of these factors were adjusted and taken into account in the studies included in our meta-analysis, which could contribute to a superficially robust association between smoking and metabolic syndrome. Nevertheless, all included studies have adjusted for age and most of them have adjusted for a wide range of potential confounders (9 of the 13 studies adjusted ≥5 confounders).

In conclusion, findings of the present meta-analysis of prospective cohort studies suggest that active smoking is associated with higher risk of metabolic syndrome. The conclusion has a far-reaching significance for public health, especially in countries of high smoking prevalence and high incidence of metabolic syndrome. Further investigations, both epidemiological and mechanistic, are needed to establish the extent to which the association can be explained by a causal link and whether smoking cessation can prevent occurrence and development of metabolic syndrome.

## References

[1] World Health Organization (2011) Tobacco Free Initiative: Why Is Tobacco a Public Health Priority. World Health Organization website. Available at: http://www.who.int/tobacco/health_priority/en/. Accessed 2012 Sep 23.

[2] International Diabetes Foundation (2006) The IDF consensus worldwide definition of the metabolic syndrome. Brussels, Belgium: IDF Communications.

[3] Balhara YP (2012) Tobacco and metabolic syndrome. Indian J Endocrinol Metab 16: 81–87. 22276256 10.4103/2230-8210.91197 PMC3263202

[4] Cena H, Fonte ML, Turconi G (2011) Relationship between smoking and metabolic syndrome. Nutrition Reviews 69: 745–753. 22133198 10.1111/j.1753-4887.2011.00446.x

[5] Onat A, Ozhan H, Esen AM, Albayrak S, Karabulut A, et al (2007) Prospective epidemiologic evidence of a “protective” effect of smoking on metabolic syndrome and diabetes among Turkish women–without associated overall health benefit. Atherosclerosis 193: 380–388. 16926017 10.1016/j.atherosclerosis.2006.07.002

[6] Stroup DF, Berlin JA, Morton SC, Olkin I, Williamson GD, et al (2000) Meta-analysis of observational studies in epidemiology: a proposal for reporting. Meta-analysis Of Observational Studies in Epidemiology (MOOSE) group. JAMA 283: 2008–2012. 10789670 10.1001/jama.283.15.2008

[7] Huxley RR, Woodward M (2011) Cigarette smoking as a risk factor for coronary heart disease in women compared with men: a systematic review and meta-analysis of prospective cohort studies. Lancet 378: 1297–1305. 21839503 10.1016/S0140-6736(11)60781-2

[8] GA Wells BS, D O Connell, J Peterson, V Welch, M Losos, et al.. (2004) The Newcastle-Ottawa Scale (NOS) for assessing the quality of nonrandomised studies in meta-analyses.

[9] Higgins JP, Thompson SG (2002) Quantifying heterogeneity in a meta-analysis. Stat Med 21: 1539–1558. 12111919 10.1002/sim.1186

[10] Higgins JP, Thompson SG, Deeks JJ, Altman DG (2003) Measuring inconsistency in meta-analyses. BMJ 327: 557–560. 12958120 10.1136/bmj.327.7414.557 PMC192859

[11] DerSimonian R, Laird N (1986) Meta-analysis in clinical trials. Control Clin Trials 7: 177–188. 3802833 10.1016/0197-2456(86)90046-2

[12] Woolf B (1955) On estimating the relation between blood group and disease. Ann Hum Genet 19: 251–253. 14388528 10.1111/j.1469-1809.1955.tb01348.x

[13] Kim BJ, Kim BS, Sung KC, Kang JH, Lee MH, et al (2009) Association of smoking status, weight change, and incident metabolic syndrome in men: a 3-year follow-up study. Diabetes Care 32: 1314–1316. 19389815 10.2337/dc09-0060 PMC2699708

[14] Nakanishi N, Takatorige T, Suzuki K (2005) Cigarette smoking and the risk of the metabolic syndrome in middle-aged Japanese male office workers. Ind Health 43: 295–301. 15895844 10.2486/indhealth.43.295

[15] Zhu Y, Zhang M, Hou X, Lu J, Peng L, et al (2011) Cigarette smoking increases risk for incident metabolic syndrome in Chinese men-Shanghai diabetes study. Biomed Environ Sci 24: 475–482. 22108412 10.3967/0895-3988.2011.05.004

[16] Kim SG, Lim HS, Cheong HK, Kim CS, Seo HJ (2007) Incidence and risk factors of insulin resistance syndrome in 20-59 year-old Korean male workers. J Korean Med Sci 22: 968–972. 18162708 10.3346/jkms.2007.22.6.968 PMC2694631

[17] Holme I, Tonstad S, Sogaard AJ, Larsen PG, Haheim LL (2007) Leisure time physical activity in middle age predicts the metabolic syndrome in old age: results of a 28-year follow-up of men in the Oslo study. BMC Public Health 7: 154. 17625024 10.1186/1471-2458-7-154 PMC1947967

[18] Wannamethee SG, Shaper AG, Whincup PH (2006) Modifiable lifestyle factors and the metabolic syndrome in older men: Effects of lifestyle changes. J Am Geriatr Soc 54: 1909–1914. 17198498 10.1111/j.1532-5415.2006.00974.x

[19] Kawada T, Otsuka T, Inagaki H, Wakayama Y, Li Q, et al (2010) Association of smoking status, insulin resistance, body mass index, and metabolic syndrome in workers: A 1-year follow-up study. Obesity Research & Clinical Practice 4: e163–e169. 10.1016/j.orcp.2009.12.004 24345659

[20] Carnethon MR, Loria CM, Hill JO, Sidney S, Savage PJ, et al (2004) Risk factors for the metabolic syndrome: the Coronary Artery Risk Development in Young Adults (CARDIA) study, 1985-2001. Diabetes Care 27: 2707–2715. 15505009 10.2337/diacare.27.11.2707

[21] Yang FY, Wahlqvist ML, Lee MS (2008) Body mass index (BMI) as a major factor in the incidence of the metabolic syndrome and its constituents in unaffected Taiwanese from 1998 to 2002. Asia Pac J Clin Nutr 17: 339–351. 18586657

[22] Wilsgaard T, Jacobsen BK (2007) Lifestyle factors and incident metabolic syndrome. The Tromso Study 1979–2001. Diabetes Res Clin Pract 78: 217–224. 17448561 10.1016/j.diabres.2007.03.006

[23] Li Y, Yatsuya H, Iso H, Tamakoshi K, Toyoshima H (2010) Incidence of metabolic syndrome according to combinations of lifestyle factors among middle-aged Japanese male workers. Prev Med 51: 118–122. 20451548 10.1016/j.ypmed.2010.04.016

[24] Puustinen PJ, Koponen H, Kautiainen H, Mantyselka P, Vanhala M (2011) Psychological distress predicts the development of the metabolic syndrome: a prospective population-based study. Psychosom Med 73: 158–165. 21148808 10.1097/PSY.0b013e3182037315

[25] Woodward M, Lam TH, Barzi F, Patel A, Gu D, et al (2005) Smoking, quitting, and the risk of cardiovascular disease among women and men in the Asia-Pacific region. Int J Epidemiol 34: 1036–1045. 15914503 10.1093/ije/dyi104

[26] Martiniuk AL, Lee CM, Lam TH, Huxley R, Suh I, et al (2006) The fraction of ischaemic heart disease and stroke attributable to smoking in the WHO Western Pacific and South-East Asian regions. Tob Control 15: 181–188. 16728748 10.1136/tc.2005.013284 PMC2564655

[27] Strauss U, Schubert R, Jung S, Mix E (1998) K+ currents of encephalitogenic memory T cells decrease with encephalitogenicity while interleukin-2 (IL-2) receptor expression remains stable during IL-2 dependent cell expansion. Receptors Channels 6: 73–87. 9664624

[28] Hill AB (1965) The Environment and Disease: Association or Causation? Proc R Soc Med 58: 295–300. 14283879 10.1177/003591576505800503 PMC1898525

[29] He Y, Lam TH, Jiang B, Wang J, Sai X, et al (2009) Combined effects of tobacco smoke exposure and metabolic syndrome on cardiovascular risk in older residents of China. J Am Coll Cardiol 53: 363–371. 19161888 10.1016/j.jacc.2008.08.073

[30] Willi C, Bodenmann P, Ghali WA, Faris PD, Cornuz J (2007) Active smoking and the risk of type 2 diabetes: a systematic review and meta-analysis. JAMA 298: 2654–2664. 18073361 10.1001/jama.298.22.2654

[31] Williamson DF, Madans J, Anda RF, Kleinman JC, Giovino GA, et al (1991) Smoking cessation and severity of weight gain in a national cohort. N Engl J Med 324: 739–745. 1997840 10.1056/NEJM199103143241106

[32] Wilkins JN, Carlson HE, Van Vunakis H, Hill MA, Gritz E, et al (1982) Nicotine from cigarette smoking increases circulating levels of cortisol, growth hormone, and prolactin in male chronic smokers. Psychopharmacology (Berl) 78: 305–308. 6818588 10.1007/BF00433730

[33] Winkelmann BR, Boehm BO, Nauck M, Kleist P, Marz W, et al (2001) Cigarette smoking is independently associated with markers of endothelial dysfunction and hyperinsulinaemia in nondiabetic individuals with coronary artery disease. Curr Med Res Opin 17: 132–141. 11759183

[34] Bigazzi R, Bianchi S (2007) Insulin resistance, metabolic syndrome and endothelial dysfunction. J Nephrol 20: 10–14. 17347967

[35] Albanes D, Jones DY, Micozzi MS, Mattson ME (1987) Associations between smoking and body weight in the US population: analysis of NHANES II. Am J Public Health 77: 439–444. 3493709 10.2105/ajph.77.4.439 PMC1646954

[36] Gordon T, Kannel WB, Dawber TR, McGee D (1975) Changes associated with quitting cigarette smoking: the Framingham Study. Am Heart J 90: 322–328. 1163424 10.1016/0002-8703(75)90320-8

[37] Chiolero A, Jacot Sadowski I, Faeh D, Paccaud F, Cornuz J (2007) Association of cigarettes smoked daily with obesity in a general adult population. Obesity (Silver Spring) 15: 1311–1318. 17495208 10.1038/oby.2007.153

[38] Shimokata H, Muller DC, Andres R (1989) Studies in the distribution of body fat. III. Effects of cigarette smoking. JAMA 261: 1169–1173. 2915440

[39] Gu D, Reynolds K, Wu X, Chen J, Duan X, et al (2005) Prevalence of the metabolic syndrome and overweight among adults in China. Lancet 365: 1398–1405. 15836888 10.1016/S0140-6736(05)66375-1

[40] Frei M, Engel Brugger O, Sendi P, Reichart PA, Ramseier CA, et al (2012) Assessment of smoking behaviour in the dental setting. A study comparing self-reported questionnaire data and exhaled carbon monoxide levels. Clin Oral Investig 16: 755–60. 10.1007/s00784-011-0583-2 21717094

